# OA05 Complex polyostotic fibrous dysplasia in McCune-Albright syndrome: diagnostic and management challenges

**DOI:** 10.1093/rap/rkag113.005

**Published:** 2026-11-03

**Authors:** Lih Yin Chong, Anupama Nandagudi

**Affiliations:** Basildon University Hospital, Basildon, United Kingdom; Basildon University Hospital, Basildon, United Kingdom; Anglia Ruskin University, Chelmsford, United Kingdom

## Abstract

**Introduction:**

Fibrous dysplasia is a rare bone disorder in which normal bone is replaced by fibrous tissue, leading to structural weakness and deformity. It may present as monostotic or polyostotic disease and can occur in association with McCune-Albright syndrome. Clinical manifestations vary depending on skeletal involvement and disease burden. Management remains challenging, particularly in young patients with persistent pain and recurrent fractures. We present a case of complex polyostotic fibrous dysplasia associated with McCune-Albright syndrome, highlighting the clinical course, psychosocial impact, and therapeutic response to bisphosphonate treatment.

**Case description:**

A 21-year-old woman (born in 2005) was diagnosed with fibrous dysplasia at the age of 13. She sustained a right fibular fracture while ice skating at age 10. Persistent right leg pain 2 years later prompted an MRI of the right lower leg that revealed focal lesions suggestive of polyostotic fibrous dysplasia, and bone scintigraphy confirmed increased tracer uptake in the proximal left femoral shaft and upper left tibial shaft. She experienced multiple fractures during adolescence, including a toe fracture at age 12 and a nasal fracture at 15. Despite stable radiographic findings, persistent pain led to a multidisciplinary review at 16, and risedronate therapy was initiated without symptomatic improvement. She was subsequently diagnosed with obsessive-compulsive disorder, anxiety, tics, and psychogenic non-epileptic seizures. At 17, hypermobility syndrome was identified. At 18, endocrinological evaluation confirmed McCune-Albright syndrome, with a café-au-lait spot on her right arm and mildly elevated FGF23 levels. Pamidronate infusions commenced at age 19 resulted in marked symptomatic improvement, with pain becoming infrequent and non-daily. Treatment was paused at age 21 upon confirmation of pregnancy. Serial imaging demonstrated stable disease throughout follow-up.

**Discussion:**

This case demonstrates the important clinical features, investigation, psychosocial aspects, and management of fibrous dysplasia in association with McCune-Albright syndrome and pregnancy.

Recurrent fractures and persistent pain in childhood prompted imaging that confirmed extensive polyostotic involvement, underscoring the diagnostic value of MRI and bone scintigraphy Managing fibrous dysplasia remains challenging because the treatment focuses on improving quality of life and reducing symptom burden. Clinicians frequently overlook the psychosocial and functional limitations that arise from treatment, which can significantly impact patients’ daily lives. Treatment with risedronate did not provide significant benefit in this patient. In contrast, intravenous pamidronate was associated with marked reduction in pain frequency and symptom burden. This suggests that intravenous bisphosphonate treatment may be more effective in selected patients with significant symptomatic disease.

Pregnancy introduces therapeutic uncertainty, as bisphosphonate safety data remain limited, warranting cautious individualized management.

Coexisting hypermobility and psychological comorbidities further complicate pain assessment and necessitate multidisciplinary care. This case emphasizes the importance of holistic management encompassing physical, psychological, and reproductive health considerations.

**Key learning points:**

**Early recognition**: Recurrent fractures and persistent bone pain in children and adolescents should prompt evaluation for underlying bone pathology. Even trauma-related fractures may reveal systemic disease when accompanied by ongoing pain.

**Longitudinal monitoring:** Imaging modalities such as MRI and bone scintigraphy are vital for diagnosis and disease mapping. However, stable radiological findings may coexist with persistent symptoms, highlighting the need for clinical correlation and regular follow-up.

**Psychosocial care:** Chronic pain and physical limitations can lead to significant psychological distress. Addressing mental health through integrated care is essential to improve overall outcomes.

**Therapeutic insight:** Intravenous bisphosphonates may offer superior symptom relief compared to oral formulations in selected patients.

**Pregnancy considerations:** Bisphosphonate use during pregnancy remains an area of uncertainty, underscoring the need for further research and individualized risk-benefit assessment.

